# X-ray diffraction from dislocation half-loops in epitaxial films

**DOI:** 10.1107/S160057672400089X

**Published:** 2024-02-23

**Authors:** Vladimir M. Kaganer

**Affiliations:** a Paul-Drude-Institut für Festkörperelektronik, Hausvogteiplatz 5-7, 10117 Berlin, Germany; Ecole National Supérieure des Mines, Saint-Etienne, France

**Keywords:** epitaxial films, strain relaxation, misfit dislocations, threading dislocations

## Abstract

X-ray diffraction from dislocation half-loops consisting of a misfit segment with two threading arms extending from it to the surface is calculated by the Monte Carlo method. The diffraction profiles and reciprocal space maps are controlled by the ratio of the total lengths of the misfit and the threading segments of the half-loops. A continuous transformation from the diffraction characteristic of misfit dislocations to that of threading dislocations with increasing thickness of epitaxial film is studied. Diffraction from dislocations with edge- and screw-type threading arms is considered and the contributions of the two types of dislocations are compared.

## Abstract 2

X-ray diffraction from dislocation half-loops consisting of a misfit segment with two threading arms extending from it to the surface is calculated by the Monte Carlo method.

## 1. Introduction

Misfit dislocations are the most common mode of strain relaxation in epitaxial films (Fitzgerald, 1991 ▸; Hull & Bean, 1992 ▸; Jain *et al.*, 1997 ▸; Bolkhovityanov *et al.*, 2001 ▸). Since the dislocation lines cannot terminate inside a crystal, a misfit dislocation is accompanied by threading arms that extend to the surface (or terminate at an incoherent boundary; we do not consider this case here). The glide of the threading arm under the action of epitaxial strain is the most prominent mechanism of strain relaxation (Matthews & Blakeslee, 1974 ▸). Threading dislocations passing through the active region of a heteroepitaxial structure lead to degradation of its electronic properties, whereas misfit dislocations, if located at the interface of a buffer layer below the active region, may have no negative effect. Therefore, a separate determination of misfit and threading dislocations is of primary interest in the characterization of heterostructures for electronic and optoelectronic applications. The density of threading dislocations can be very low if the dislocations glide over long distances, up to the entire length of the sample. At the other extreme, epitaxial gallium nitride is a well known example of a crystal with high threading dislocation densities (Bennett, 2010 ▸).

Shifts in the positions of the X-ray diffraction (XRD) peaks due to relaxation of the average strain by misfit dislocations are commonly used to detect strain relaxation and the corresponding misfit dislocation density (Heinke *et al.*, 1994 ▸). Dislocations also cause inhomogeneous strain, leading to additional diffuse scattering at low dislocation densities and to a broadening of the X-ray peaks at high dislocation densities. The interpretation of the diffraction peak profiles is not as straightforward as that of the mean strain due to dislocations, since the positions of the dislocations may be correlated for kinetic or energetic reasons. The elastic energy of a dislocation array is reduced when misfit dislocations reduce fluctuations in the mean distances between dislocations, from a random to a more periodic arrangement. Threading dislocations reduce the elastic energy when dislocations with opposite Burgers vectors are closer together to compensate for long-range strain.

The theory of XRD from misfit (Kaganer *et al.*, 1997 ▸) and threading (Kaganer *et al.*, 2005 ▸) dislocations takes these correlations into account and shows that the diffraction peak profiles are sensitive to them. Scattering from misfit dislocations cannot be neglected even in situations where the threading dislocations dominate. Reciprocal space maps of GaN films several micrometres thick, where threading dislocations are expected to dominate, also showed a significant scattering from misfit dislocations (Kopp *et al.*, 2014*a* ▸,*b* ▸). In these studies, misfit and threading dislocations were considered as two separate dislocation arrays uncorrelated with each other.

It is more appropriate to model the dislocation distribution by dislocation half-loops consisting of a misfit segment with two threading arms extending from it to the surface. The two threading segments have opposite displacement fields, corresponding to opposite directions of the dislocation lines when the Burgers vector is kept constant along the half-loop. Equivalently, the two threading segments can be considered to have opposite Burgers vectors if the dislocation line directions are taken to be the same. These threading dislocations screen the strain fields from each other and provide a model of the dislocation correlations that reduce the elastic energy of the film (Kaganer & Sabelfeld, 2010 ▸). By varying the relative lengths of the misfit and threading segments, one can go from the limiting case of misfit dislocations to the opposite limit of threading dislocations. The elastic field of a dislocation half-loop is quite complicated (see the supporting information) and the diffraction from the half-loops cannot be studied analytically. However, the XRD from a statistical distribution of defects with known elastic fields can be calculated by the Monte Carlo method (Kaganer & Sabelfeld, 2009 ▸, 2010 ▸).

The aim of the present work is to model the XRD from dislocation half-loops. We follow the transformation of the reciprocal space maps and the diffraction profiles with increasing film thickness while keeping the dislocation density constant. In this way the change from the diffraction pattern characteristic of misfit dislocations to that of threading dislocations can be analyzed. We show that the parameter controlling this transformation is the ratio of the total lengths of misfit and threading dislocations, or equivalently, the ratio of the mean length of the misfit segment to the film thickness. We find that this transformation is rather smooth and also depends on the inclination of the actual diffraction vector to the surface. We compare the effects of the half-loops with the edge and screw dislocation types of the threading arms, and find that they both contribute to the symmetric Bragg reflections.

## 2. Monte Carlo simulation of X-ray diffraction

**Figure 1:**
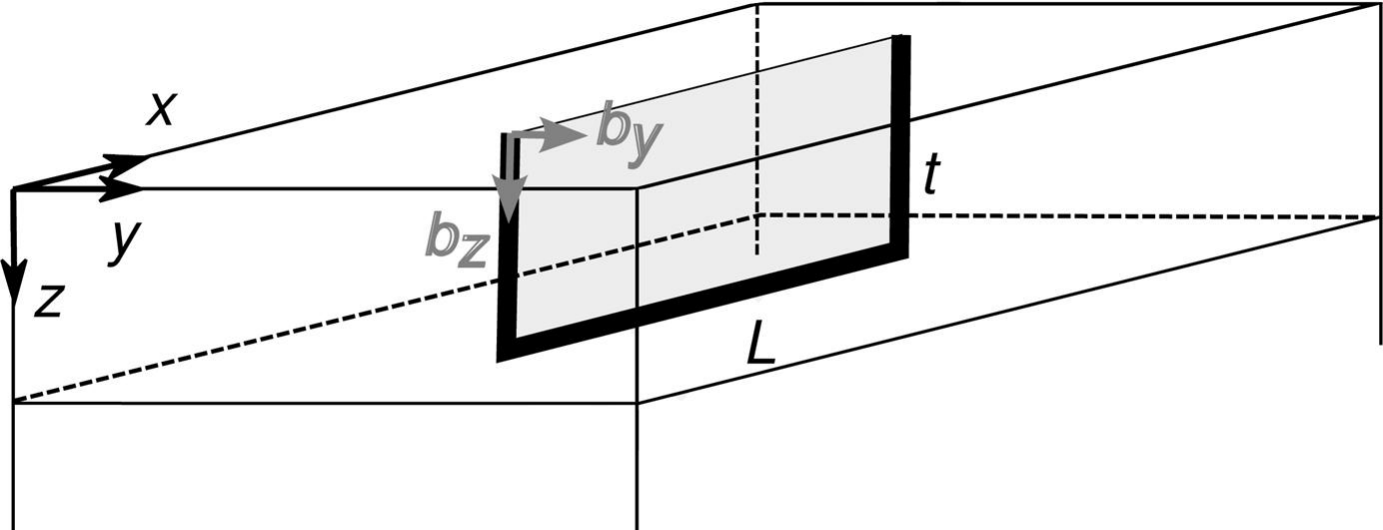
Geometry of an epitaxial film with a dislocation half-loop.

We study the XRD from the dislocation half-loops sketched in Fig. 1 ▸. Threading arms are assumed to be straight and perpendicular to the film surface. Two types of dislocations are considered. Dislocations with edge-type threading arms (denoted *b_y_* in Fig. 1 ▸) have Burgers vectors normal to the half-loop plane. Such half-loops correspond to the insertion (or removal, depending on the sense of the mismatch) of a rectangular piece of an extra atomic plane, bound by the dislocation line and shaded in Fig. 1 ▸. The second type of dislocation has screw-type threading dislocation arms (denoted *b_z_* in Fig. 1 ▸). Their misfit segments provide a local tilt of the film. For these half-loops, Burgers vectors with opposite signs are taken with equal probability, so there is no net tilt of the film.

We take the density of the threading dislocation arms ρ_T_ and the mean length of the misfit segment *L* as two parameters that characterize the dislocation ensemble. The misfit dislocation density is therefore ρ_M_ = *L*ρ_T_/2, since each half-loop has two threading arms. We note that the threading dislocation density ρ_T_ and the misfit dislocation density ρ_M_ have different dimensionalities. The threading dislocation density is the number of threading dislocations per unit area of the surface, or more generally, the total length of the threading dislocations per unit volume. The misfit dislocation density is the number of dislocations per unit length of the interface, or more generally, the total length of the dislocation lines per unit area of the interface.

A parameter that controls the relative contributions of misfit and threading dislocations is the ratio *L*/*t* of the mean length of the misfit segment *L* to the film thickness *t*. One can also compare the total length of misfit dislocations per unit area of the interface ρ_M_ with that of threading dislocations ρ_T_ *t*, since the length of each threading segment is *t*. Given the definition of ρ_M_ above, this ratio is simply *L*/2*t*. Another parameter of the dislocation array is the dimensionless parameter *M* introduced by Wilkens (1970*a* ▸, 1976 ▸) to characterize the screening of the dislocation strain by the surrounding dislocations. It is equal to the ratio of the mean distance *L* between threading dislocations with opposite Burgers vectors (assuming the same dislocation line directions of the threading segments) to the mean distance between threading dislocations 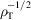 , so that 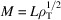 .

The Monte Carlo simulations below are performed for a GaN{0001} epitaxial film as an example. The positions of the dislocation half-loops are random and uncorrelated. The lengths *L* of the misfit segments have a lognormal distribution with the standard deviation *L*/2. The misfit segments of the half-loops run in three equivalent 〈1100〉 directions with equal probability. The length of the Burgers vector of a half-loop with edge threading arms *b_y_* is *a* = 0.319 nm, while that of the half-loop with screw threading arms *b_z_* is *c* = 0.518 nm. The displacement field of a half-loop, satisfying the elastic boundary conditions of the free surface, is constructed from the displacement field of an angular dislocation near the free surface (Comninou & Dundurs, 1975 ▸) and that of a dislocation normal to the surface (Lothe, 1992 ▸). Details of the construction and the analytical expressions for all components of the displacements are given in the supporting information.

The choice of Poisson’s ratio to model dislocations in GaN is somewhat ambiguous. In strain relaxation problems for elastically anisotropic epitaxial films, the Poisson ratio is commonly chosen to give the same vertical strain as in the isotropic approximation. For GaN(0001), this requirement gives ν = *c*_13_/(*c*_13_ + *c*_33_), where *c_ij_* are the anisotropic elastic moduli. The value ν = 0.21 is obtained using the elastic moduli of GaN given by Polian *et al.* (1996 ▸). The measured values of ν vary from 0.15 to 0.23 (Moram & Vickers, 2009 ▸). On the other hand, the strain field of a straight edge dislocation with a 〈0001〉 dislocation line direction in an anisotropic hexagonal crystal coincides with the isotropic solution when the Poisson ratio is taken to be ν_h_ = *c*_12_/(*c*_12_ + *c*_11_) (Belov, 1992 ▸). Using the elastic moduli of GaN (Polian *et al.*, 1996 ▸), Poisson’s ratio is ν_h_ = 0.27. We use the latter value in the Monte Carlo simulations below to obtain a better representation of the strain fields of the threading dislocation arms.

The diffracted intensity is a Fourier transform of the correlation function 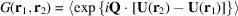 to reciprocal space. Here **r**_1_ and **r**_2_ are the coordinates of two points inside the crystal, **U**(**r**) is the total displacement produced by all dislocations (equal to the sum of the dis­placement fields of individual dislocations due to linear elasticity) calculated at these two points, and **Q** is the diffraction vector. The statistical average 〈…〉 over the dislocation ensemble and the Fourier transform can be performed simultaneously in one and the same Monte Carlo integration (Kaganer & Sabelfeld, 2009 ▸). This integration is time consuming, especially when dislocation densities are large and low intensities at asymptotes are of interest: the integration is a summation of complex numbers of modulus 1 to finally obtain a real number which is much less than 1.

When the mean-squared strain in a crystal is large, only correlations between closely spaced points **r**_1_ and **r**_2_ are impor­tant. The expansion 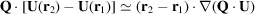 allows us to reduce the calculation of the X-ray intensity to the calculation of the probability density of the respective distortion components (Stokes & Wilson, 1944 ▸). This approximation, without a reference to Stokes and Wilson, is the basis of the theory of XRD from crystals with dislocations by Krivoglaz & Ryaboshapka (1963*a* ▸,*b* ▸) and Wilkens (1970*a* ▸,*b* ▸, 1976 ▸). In particular, this approximation, applied to the strain field of a single dislocation, gives the well known asymptotic law for the X-ray intensity 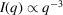 at large *q*.

Kaganer & Sabelfeld (2014 ▸) studied the applicability limits of the Stokes–Wilson (SW) approximation in Monte Carlo simulations of the diffraction from various arrays of parallel dislocations. It was shown that the SW approximation is applicable as long as long-range order is absent, *i.e.* its manifestation as a coherent (resolution-limited) peak is not seen in the diffraction profile. For dislocations in a bulk crystal, the coherent peak arises when the dislocations form dipoles with a small distance between dislocations in the dipole compared with the distance between the dipoles. This situation corresponds to the Wilkens parameter *M* < 1. The accuracy of the SW approximation increases as *M* > 1 is increased. For epitaxial films, the coherent peak arises when the mean distance between misfit dislocations is larger than the film thickness, or the misfit dislocations are almost periodic. In these cases, the calculation of the diffraction profile as a probability density distribution of distortions fails, and a calculation with the difference of displacements must be performed.

**Figure 2:**
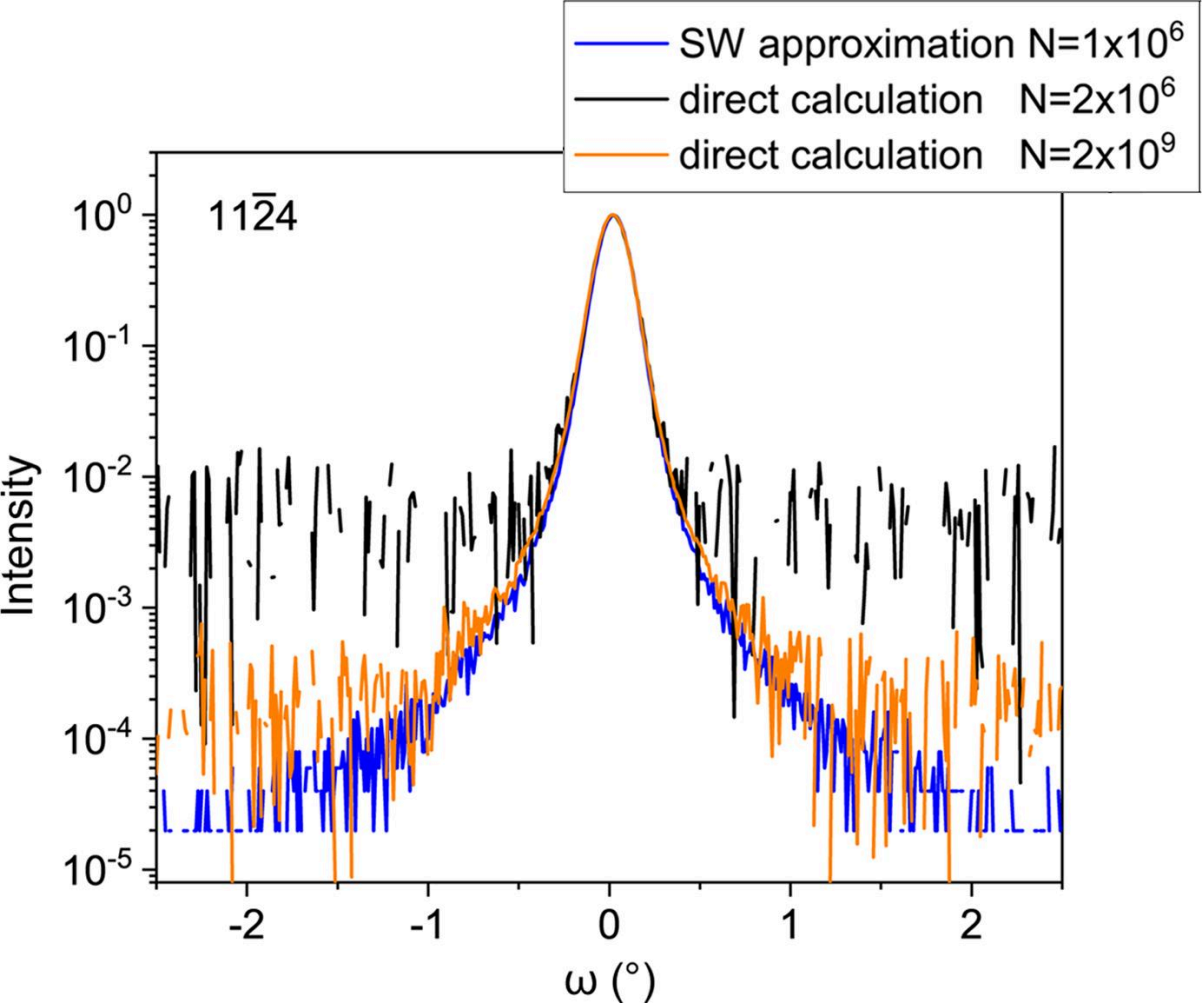
XRD profile of the 1124 reflection in skew geometry for dislocation half-loops with edge threading arms. Threading dislocation density ρ_T_ = 1 × 10^10^ cm^−2^, mean length of the misfit segments *L* = 1 µm, film thickness *t* = 1 µm. The calculation in the SW approximation (blue line) is compared with the direct calculation by performing a Fourier transform of the correlation function 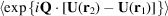 . The black curve is a calculation that takes the same computational time as that in the SW approximation, whereas calculation of the orange curve took 10^3^ times longer.

**Figure 3:**
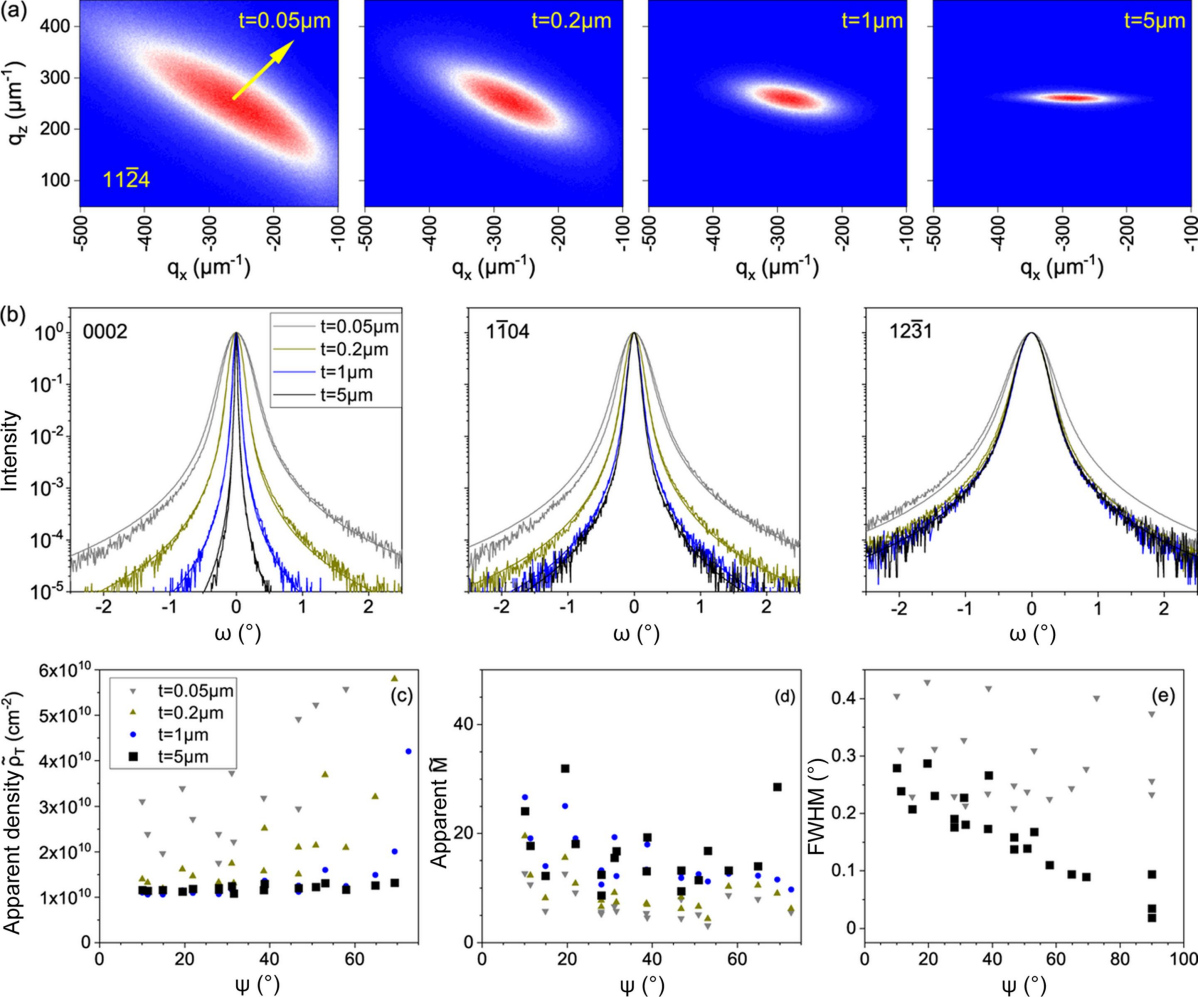
Monte Carlo calculation of the XRD from dislocation half-loops with edge threading arms. Threading dislocation density ρ_T_ = 1 × 10^10^ cm^−2^, mean length of the misfit segments *L* = 1 µm. (*a*) Reciprocal space maps for the 1124 reflection for different epitaxial layer thicknesses. The diffraction vector is indicated by an arrow on the left map. (*b*) Diffraction peak profiles in skew geometry. The noisy lines are Monte Carlo simulations, and the smooth curves are fits that treat the diffraction intensity as due only to threading dislocations. (*c*) Apparent density of threading dislocations 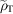 and (*d*) apparent values 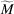 of the Wilkens parameter obtained in these fits. (*e*) FWHM of the diffraction profiles of the reflections. Ψ is the angle between the reflection vector and the crystal surface.

**Figure 4:**
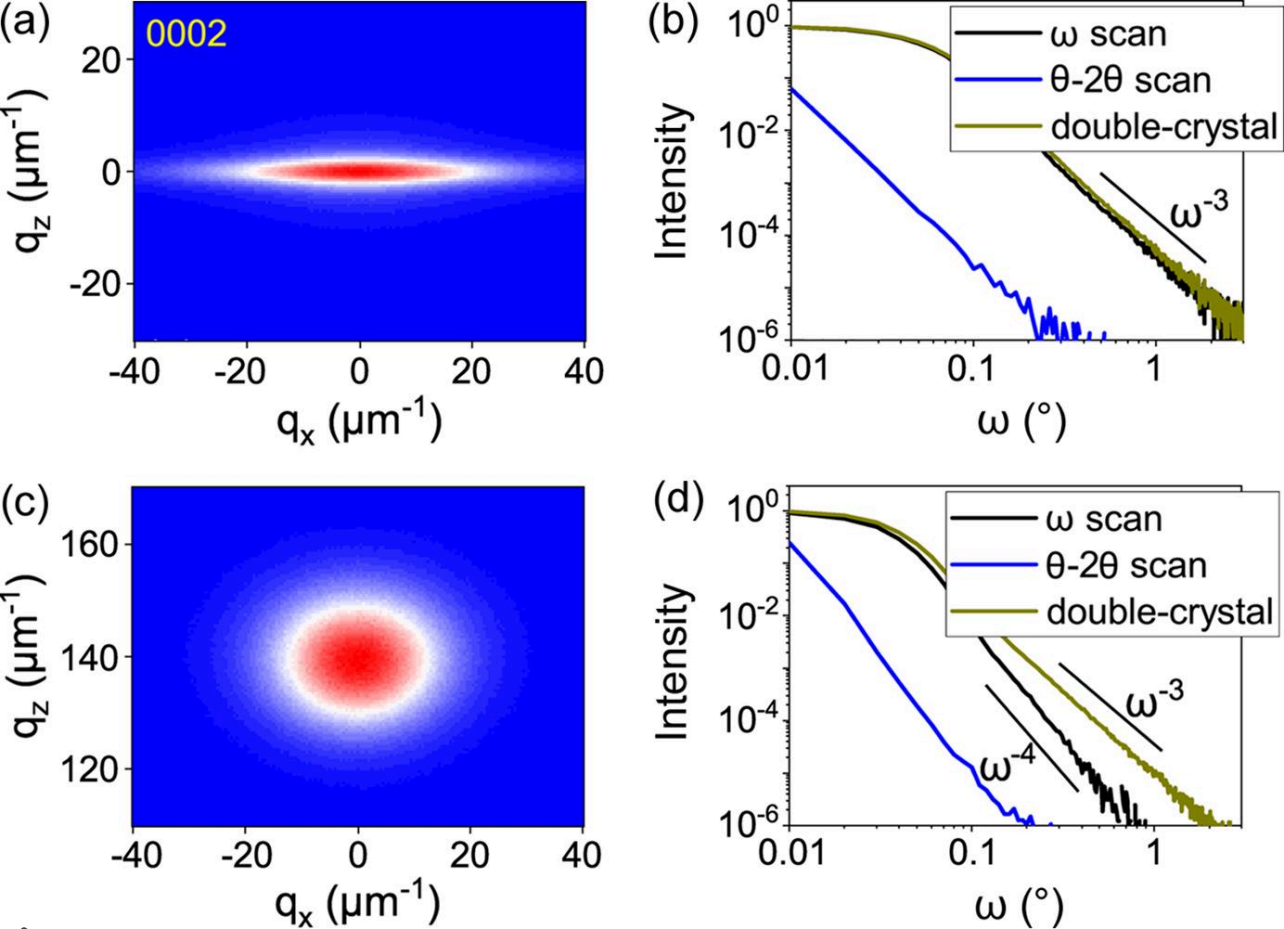
Reciprocal space maps for the symmetric 0002 Bragg reflection from dislocation half-loops with (*a*) screw threading arms, ρ_T_ = 1 × 10^9^ cm^−2^; and (*c*) edge threading arms, ρ_T_ = 1 × 10^10^ cm^−2^. The mean length of the misfit segments is *L* = 1 µm and the film thickness is *t* = 1 µm. The ω and θ–2θ triple-crystal scans through the maps, as well as the double-crystal scans, are shown in (*b*) and (*d*) in log–log scale.

Calculation of the diffraction profile in the SW approximation avoids the summation of oscillating complex terms and drastically reduces the computation time. Fig. 2 ▸ compares the calculation of an XRD profile in the SW approximation and directly by Fourier transform of the correlation function 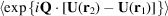 . Details of the calculations are described below and in the supporting information. The calculation in the SW approximation (blue line) is an average over *N* = 1 × 10^6^ dislocation arrangements, which took approxi­mately 9 min in our computational setup described below. A direct calculation using the difference of displacements with an average over *N* = 2 × 10^6^ dislocation arrangements (black line) took the same computation time, since the calculation of the *x* and *z* strain components by finite differences requires twice the number of displacement calculations. The accuracy achieved in the calculations is evident from the noise levels of the respective curves: the accuracy of the strain-based calculation is at least 10^2^ higher. A 10^3^ times longer calculation using the difference of displacements (orange line), which took one week in our computational setup, shows good agreement with the calculation in the SW approximation, but still does not reach its accuracy. Therefore, all calculations in Figs. 3 and 4 are performed using the SW approximation.

The relevant strain components in the SW approximation depend on the diffraction geometry. Stokes & Wilson (1944 ▸) developed their approximation for powder diffraction, in which case the normal strain in the direction of the diffraction vector is involved. The corresponding components for the reciprocal space maps and skew diffraction geometry of single crystals are discussed in detail in the supporting information. Here we note that the intensity *I*(*q_x_*, *q_z_*) in the reciprocal space map is calculated as the joint probability density of the distortions 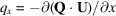 and 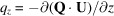 . These distortions depend on the depth *z* of the point in the epitaxial film at which they are calculated. Therefore, an integration over *z* is performed from the surface *z* = 0 to the interface *z* = *t*. As is usual for a Monte Carlo simulation, this integration does not require any additional computational effort: the point *z* is randomly and homogeneously seeded on the interval [0, *t*]. Similarly, the intensity *I*(*q*) in a double-crystal scan with an open detector, in particular a scan in skew geometry, is calculated as the probability density of the distortion 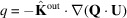 , where 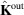 is a unit vector in the direction of the diffracted beam and the integration of the probability density over *z* is performed, as above. The wavevector value *q* is related to the angular deviation ω by *q* = *Q*ωcosθ, where θ is the Bragg angle of the actual reflection.

This Monte Carlo calculation is ideally suited to parallel computing since each realization of the random dislocation distribution can be computed independently and the partial sums obtained on different processors can be added at the end. We use the coarray extension to Fortran, which was added to the language standard in 2008. In practice, the parallel computations require only a few lines of code to be modified and are executed on 128 cores of an Epyc 7763 compute server without any loss of computational efficiency.

Diffraction profiles and maps are typically computed in less than 1 min with sufficient accuracy to reveal the features of the intensity distribution. Computation times of up to several hours are used to reduce the statistical noise. Since the statistical error decreases as 1/*N* ^1/2^, where *N* is the number of repetitions, the 1 min runs are only an order of magnitude less accurate in intensity. The computation time can be reduced by choosing larger steps in the angles in the curves and wavevectors in the maps. On the other hand, most of the computation time is required for the calculation of the dislocation displacements using the analytical formulae presented in the supporting information, which leaves very little room for improvement. The calculation requires memory for an array of the calculated intensity and an array of the coordinates of the dislocations in an actual realization of their distribution, which together do not exceed several megabytes per core.

## 3. Results

Let us consider the XRD from dislocation half-loops with edge threading arms. We assume a threading dislocation density ρ_T_ = 1 × 10^10^ cm^−2^ and a mean length of the misfit segments *L* = 1 µm. Fig. 3 ▸(*a*) shows the transformation of the 1124 reciprocal space maps with increasing film thickness. We note first that the position of the intensity maximum 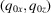 does not depend on the film thickness. Each dislocation half-loop provides a rectangular cut of size *L* × *t* (see the shaded rectangle in Fig. 1 ▸), where an extra lattice plane of thickness *b*_e_ is inserted. Here the edge component of the Burgers vector normal to the half-loop plane is denoted by *b*_e_, instead of *b_y_* in Fig. 1 ▸, to account for half-loops of different orientations. When this area is divided by the volume per half-loop, the in-plane strain is 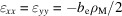 . Hence, the position of the X-ray peak is 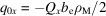 and, due to the Poisson effect, 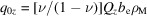 , where *Q_x_* and *Q_z_* are the lateral and the normal components of the scattering vector, respectively. The peak positions in Fig. 3 ▸(*a*) correspond to these formulae (the substrate peak is taken at *q_x_* = *q_z_* = 0). To obtain these peak positions in Monte Carlo simulations, one has to increase the lateral size of the simulated region proportional to the film thickness. The simulations in Fig. 3 ▸(*a*) are performed with the lateral size of 25*t*, which requires a corresponding increase in computing time for thick films. However, the lateral size of the simulated region affects only the peak positions; the sizes and shapes of the intensity spots are only slightly affected.

For a thickness *t* = 0.05 µm, which is small compared with the misfit segment length, the misfit dislocations dominate the diffraction. The reciprocal space map has the same features as that of infinitely long misfit dislocations (Kaganer *et al.*, 1997 ▸). It is extended in the direction almost perpendicular to the direction of the diffraction vector, indicated by an arrow in the figure (these directions need not be exactly perpendicular to each other as this is not required by symmetry). In the opposite limit, where the thickness *t* = 5 µm is large compared with the misfit segment length, the diffraction is dominated by threading dislocation arms. Since threading dislocations are parallel straight lines in real space, their diffraction pattern in reciprocal space is a disk perpendicular to the dislocation line (Kopp *et al.*, 2014*a* ▸,*b* ▸). A section of the disk through the scattering plane gives the horizontal streak in the map. The maps in Fig. 3 ▸(*a*) show a gradual transition from one limit to the other. At thickness *t* = 0.2 µm, five times smaller than the misfit segment length, the diffraction pattern already differs from that for misfit dislocations. At thickness *t *= 5 µm, five times larger than the misfit segment length, there is still a finite width of the intensity spot in the *q_z_* direction.

Fig. 3 ▸(*b*) shows diffraction profiles in skew geometry (Srikant *et al.*, 1997 ▸; Sun *et al.*, 2002 ▸; Kaganer *et al.*, 2005 ▸) for the same film thicknesses and for three reflections, a symmetric reflection (0002, left), a slightly asymmetric reflection (1104, middle) and a highly asymmetric reflection (1231, right). The intensities calculated by the Monte Carlo method are seen as noisy lines, while smooth lines of the same colors are the fits discussed below. Let us start the analysis with the symmetric reflection. Since straight edge dislocations in an infinite medium produce strain only in the plane normal to the dislocation line, it is expected that edge threading dislocations will not cause any broadening of the symmetric reflections. However, the plot in Fig. 3 ▸(*b*) shows that the total effect of the strain field of the misfit segment and the strain due to stress relaxation at the free surface of the threading segments of the half-loop give rise to a diffraction peak broadening even at a thickness of 5 µm.

In the usual treatment of broadening of symmetric reflections as a manifestation of the screw dislocations, this broadening would be interpreted as a density of screw dislocations. The smooth lines in the plots of Fig. 3 ▸(*b*) are the fits proposed by Kaganer *et al.* (2005 ▸) for threading dislocations. They include two parameters, the dislocation density and the length of the strain field screening (or the dimensionless parameter *M*). An application of the formulae derived for straight threading dislocations for dislocation half-loops is not justified. These fits only show apparent dislocation densities obtained when the diffraction profiles are treated as the result of threading dislocations. The apparent density of screw threading dislocations obtained in the fit of the 0002 reflection for a film thickness of 5 µm is 1.1 × 10^8^ cm^−2^. The apparent density of screw dislocations increases with decreasing film thickness, as can be seen in the plots, and reaches 6.5 × 10^9^ cm^−2^ for a film thickness of 0.05 µm.

At the opposite extreme of the highly asymmetric 1231 reflection in the right plot of Fig. 3 ▸(*b*), the strain due to edge threading arms dominates. The diffraction profiles almost coincide for a film thicknesses of 0.2 µm and above. The slightly asymmetric 1104 reflection in the middle plot of Fig. 3 ▸(*b*) shows an intermediate behavior: for thicknesses less than 1 µm, the misfit segment of the half-loop makes a significant contribution.

Figs. 3 ▸(*c*) and 3 ▸(*d*) summarize the results of the fits made by the model for infinitely long edge threading dislocations (Kaganer *et al.*, 2005 ▸). These fits are represented by smooth lines in Fig. 3 ▸(*b*). A total of 19 diffraction profiles for different asymmetric reflections in skew geometry are calculated by the Monte Carlo method. The apparent density of edge threading dislocations 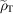 and the corresponding apparent parameter 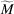 are obtained in the fits. The results for different reflections are compared by plotting these apparent parameters as a function of the angle Ψ between the diffraction vector and the film surface. Ψ = 0 corresponds to diffraction in the surface plane, and Ψ = 90° to symmetric reflections. The symmetric reflections are not included in Figs. 3 ▸(*c*) and 3 ▸(*d*) since they have been fitted to screw rather than edge threading dislocations.

The results for the film thickness of 5 µm are shown in Figs. 3 ▸(*c*) and 3 ▸(*d*) by filled squares, deliberately made larger than the symbols for the other thicknesses, as they come closest to the model of infinite threading dislocations assumed by the fits. The dislocation density obtained in the fit for this film thickness is quite close to the density of 1 × 10^10^ cm^−2^ modeled in the Monte Carlo simulations. This result confirms the consistency between the present Monte Carlo simulations and the fits with the formulae proposed by Kaganer *et al.* (2005 ▸). Fig. 3 ▸(*c*) shows that, as the thickness decreases, the misfit parts of the half-loops make progressively larger contributions. The apparent density of edge dislocations can be six times larger than the real density. It can also be seen that the apparent density systematically depends on the inclination angle Ψ of the reflection: the less asymmetric reflections give a larger apparent density. This dependence can help us to recognize the contribution of misfit dislocations.

The input value of the parameter *M* in the Monte Carlo simulations is 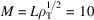 . The values 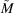 obtained in the fit are larger and show a large scatter even for the 5 µm film thickness, where the threading dislocations dominate. This result is not surprising: as discussed by Kaganer *et al.* (2005 ▸), the fit formula does not take into account the orientation factors involved in this parameter. As a result, the accuracy of the dislocation correlation determination is lower than that of the dislocation density determination. As also discussed by Kaganer & Sabelfeld (2010 ▸), the consideration of these orientation factors is a rather complicated task. In many applications, it is the dislocation density rather than the dislocation correlations that is of primary interest. Correct determination of the dislocation screening length is particularly important to determine the strain energy stored in the dislocated crystal (Borbély *et al.*, 2023 ▸). A possible way to determine the screening length is to perform a Monte Carlo simulation of the diffraction profiles of all the reflections of interest and introduce correction factors 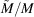 to the parameters obtained by fitting the experimental curves.

The Monte Carlo simulated profiles (noisy lines) in Fig. 3 ▸(*b*) are asymmetric compared with the fits (smooth lines) which are symmetric. The asymmetry is a consequence of the nature of the half-loops: they are all of the insertion type, corresponding to the introduction of a piece of an extra atomic plane, and not to its removal. Such ‘polarized’ dislocation distribution gives rise to a higher-order term in the correlation function (Groma *et al.*, 1988 ▸), which is not included in our fits.

Fig. 3 ▸(*e*) shows the full widths at half-maximum (FWHMs) of the peaks obtained from the Monte Carlo simulation. The data are shown for film thicknesses of 0.05 and 5 µm. The points from the intermediate thicknesses (not shown) are scattered in between. The FWHMs are used to estimate the dislocation density via the formula 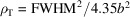 (Metzger *et al.*, 1998 ▸), which is popular because of its extreme simplicity. This formula is used for symmetric or asymmetric reflections with the length of the Burgers vector *b* equal to either the *c* or the *a* lattice parameter of GaN to obtain the densities of either screw or edge dislocations. The correct use of this formula for edge dislocations implies the use of twist, *i.e.* extrapolation of the peak widths in Fig. 3 ▸(*e*) to Ψ = 0 (Sun *et al.*, 2002 ▸).

When the threading dislocation arms are long and dominate in the scattering (filled squares), the FWHMs of the asymmetric reflections in Fig. 3 ▸(*e*) increase with the increasing inclination of the reflection (the angle Ψ decreases). The same dependence is observed in experiments (Heinke *et al.*, 2000 ▸; Sun *et al.*, 2002 ▸; Kaganer *et al.*, 2005 ▸). Extrapolation to Ψ = 0 gives a ‘twist’ of 0.3° which, according to the above formula, gives a threading dislocation density of 6 × 10^9^ cm^−2^, about half of the threading dislocation density used as input in the Monte Carlo simulations. Thus, this simple formula gives a reasonable estimate of the threading dislocation density, with some underestimation. Further Monte Carlo simulations (not presented here) show that this underestimation is systematic. The reflections for a thin epitaxial film, shown by triangles in Fig. 3 ▸(*e*), give a large scatter of the FWHMs of different reflections and, on average, a similar ‘twist’. Hence, the FWHM-based determination of the threading dislocation density gives the same underestimate. The FWHMs of the symmetric reflections, shown by the points at Ψ = 90° in Fig. 3 ▸(*e*), depend significantly on the order of the reflections. The 0002 reflection would give an apparent density of screw dislocations of 1 × 10^7^ cm^−2^ for the 5 µm-thick film and 4 × 10^9^ cm^−2^ for the 0.05 µm-thick film.

Fig. 4 ▸ shows the reciprocal space maps and the diffraction profiles for the symmetric 0002 reflection of dislocation half-loops with screw (top) and edge (bottom) threading arms. In both cases, the mean length of the misfit segments and the film thickness are taken to be the same, *L*= 1 µm and *t* = 1 µm. The dislocation densities differ by an order of magnitude; half-loops with screw threading arms of density ρ_T_ = 1 × 10^9^ cm^−2^ are compared with half-loops with edge threading arms of density ρ_T_ = 1 × 10^10^ cm^−2^. For half-loops with screw threading arms, the diffraction peak of the film in Fig. 4 ▸(*a*) coincides with that of the substrate, *q*_0*x*_ = *q*_0*z*_ = 0. For the edge threading arms in Fig. 4 ▸(*c*), the film peak is shifted in accordance with the formulae above. Note that *q*_0*z*_ is just half of that in Fig. 3 ▸(*a*), since the dislocation density is the same and the *Q_z_* component of the diffraction vector is two times smaller (0002 versus 1124).

The screw threading arms dominate in the diffraction pattern of the respective half-loops, since the displacement caused by a screw dislocation occurs along the diffraction vector. As a result, the diffraction intensity in the map of Fig. 4 ▸(*a*) is extended in the lateral direction, perpendicular to the direction of the screw arms. The scan in the *q_x_* direction in the map, which coincides with the ω scan in the symmetric reflection, collects all the diffracted intensity. Fig. 4 ▸(*b*) shows that the ω scan and the double-crystal scan almost coincide and have the expected 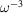 asymptote.

The edge threading arms contribute to diffraction in a symmetric Bragg reflection only due to the strain resulting from elastic relaxation at the free surface, since the displacement field of the edge threading dislocation in an infinite medium is perpendicular to the diffraction vector. The intensity in the reciprocal space map in Fig. 4 ▸(*c*) is mainly due to the misfit segments of the half-loops and extends in both the *q_x_* and the *q_z_* directions. The intensity in the ω scan shown in Fig. 4 ▸(*d*) has an 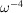 asymptote, while the additional integration in the reciprocal space for the double-crystal scan gives rise to an 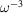 dependence.

Comparing the double-crystal scans in Figs. 4 ▸(*b*) and 4 ▸(*d*), one can see that 1 × 10^9^ cm^−2^ half-loops with screw threading arms and 1 × 10^10^ cm^−2^ half-loops with edge threading arms give very close diffraction curves. Thus, when dislocation half-loops with comparable lengths of the misfit and threading segments are present, the common assumption that the intensity in symmetric Bragg reflections is due to screw threading dislocations and the intensity in asymmetric reflections is due to edge threading dislocations is no longer valid.

## 4. Conclusions

The use of the displacement field of an angular dislocation allows the construction of arbitrary dislocation arrangements in epitaxial films, in particular dislocation half-loops. The XRD of an epitaxial film with an arbitrary density of dislocation half-loops can be calculated by the Monte Carlo method. When the diffraction profile does not possess a coherent peak, the diffraction intensity can be calculated as the probability density of the corresponding strain components, in the SW approximation. The use of this approximation allows the calculation time to be reduced by several orders of magnitude.

The shape of the double-crystal diffraction curves for half-loops is the same as that for threading dislocations. When both misfit and threading dislocations are present, a joint analysis of the double-crystal diffraction curves in skew geometry and reciprocal space maps in coplanar geometry is required to distinguish their contributions.

XRD from dislocation half-loops is controlled by the ratio of the total lengths of the misfit and the threading segments. A significant deviation from the scattering pattern of misfit dislocations is already seen in the reciprocal space maps when this ratio is 5:1, and the opposite limit of threading dislocations is not yet reached when this ratio is 1:5. The apparent density of threading dislocations obtained by fits to the formula derived for threading dislocations alone is up to six times larger than the real density of the threading segments. The apparent density obtained in this way scatters significantly depending on the reflection chosen. This scatter in density can be used to distinguish between half-loops and infinitely long threading dislocations. Another indicator that may help to distinguish between these two cases is the dependence of the FWHMs of the reflections on the angle Ψ between the reflection vector and the surface. For threading dislocations the FWHM increases as Ψ decreases. When misfit dislocations dominate, the FWHMs show a larger scattering without a systematic Ψ dependence.

For half-loops with comparable total lengths of the misfit and threading segments, the half-loops with edge and screw threading arms both contribute to the diffraction curves of symmetric Bragg reflections. The contribution of the half-loops with screw threading arms is an order of magnitude larger for comparable dislocation densities. However, since the densities of the screw threading dislocations in GaN films grown by molecular beam epitaxy are an order of magnitude smaller than those of edge dislocations, the contributions of the two dislocation types are comparable. In this case, a clear distinction between the dislocation types can be seen in the reciprocal space maps: the diffraction spot for half-loops with edge threading arms is roundish, whereas for those with screw arms it is laterally elongated.

## 5. Related literature

The following reference is cited only in the supporting information: Thomas (1993 ▸). 

## Supplementary Material

Details of the calculations. DOI: 10.1107/S160057672400089X/nb5366sup1.pdf
