# Supplementary material for: X-ray diffraction from dislocation half-loops in epitaxial films
Source: J Appl Crystallogr. 2024 Feb 23;57(Pt 2):276–83. doi: 10.1107/S160057672400089X (PMC11001416; doi:10.1107/S160057672400089X)
Supplement: Supplementary file 1 [file j-57-00276-sup1.pdf]

JOURNAL OF
APPLIED
CRYSTALLOGRAPHY

Volume 57 (2024)

Supporting information for article:

X-ray diffraction from dislocation half-loops in epitaxial films

Vladimir M. Kaganer

Supplementary Information to the paper
X-ray diffraction from dislocation half-loops in epitaxial films

Vladimir M. Kaganer
Paul-Drude-Institut für Festkörperelektronik, Hausvogteiplatz 5–7, 10117 Berlin, Germany

I. CONSTRUCTION OF THE DISPLACEMENT FIELD OF A DISLOCATION HALF-LOOP

Figure 1 shows a construction of the displacement field of a dislocation half-loop. The building blocks are an angular dislocation with one arm parallel to the surface and the other arm perpendicular to it, and a straight dislocation perpendicular to the surface. The explicit expressions for the respective displacement fields are given in Sec. II and III below. First, we construct an L-shaped dislocation consisting of a half-infinite dislocation parallel to the surface and a finite dislocation segment from it to the surface, see Fig. 1(a). It is obtained by subtracting the displacement field of the straight dislocation from that of the angular dislocation. The difference between the displacements of two such L-shaped dislocations, lying in the same plane and shifted with respect to each other, gives rise to a dislocation half-loop with the misfit dislocation segment parallel to the surface and two threading dislocation segments from it to the surface, as shown Fig. 1(b).

II. DISPLACEMENT FIELD OF AN ANGULAR DISLOCATION WITH ONE ARM PARALLEL TO THE SURFACE AND THE OTHER ARM PERPENDICULAR TO IT

Comninou and Dundurs [1] derived explicit expressions for the displacement field of an angular dislocation with one arm perpendicular to the free surface and the other arm making an arbitrary angle β to the surface. Several typos are listed by Thomas [2]. These expressions are simplified below for the case of the second dislocation arm parallel to the surface, $\beta = \pi/2$. In this case several 0/0 uncertainties have to be resolved, so using general formulae for $\beta = \pi/2$ would require some caution.

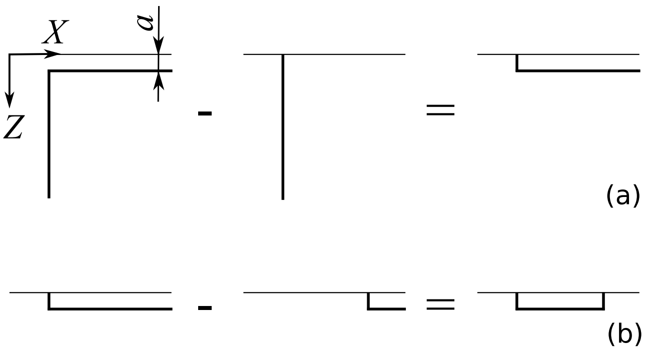

FIG. 1. Construction of the displacement field of a dislocation half-loop: (a) subtract the displacement field of a dislocation perpendicular to the surface from the displacement field of an angular dislocation to obtain an L-shaped dislocation and (b) take the difference between the displacement fields of two L-shaped dislocations shifted with respect to each other. All dislocations have the same Burgers vector.

We use a coordinate system with the origin at the surface, the X-axis parallel to the surface, the Z-axis along the inner surface normal, the dislocation lies in the XZ-plane, the distance between the surface and the dislocation arm parallel to it is a [see Fig. 1(a) and Fig. 1 in the main text of the paper]. A relation to three coordinate systems introduced by Comninou and Dundurs [1] is

$$X = y_1 = \bar{y}_1 = z_3 = -\bar{z}_3, \quad (1)$$

$$Y = y_2 = \bar{y}_2 = z_2 = \bar{z}_2, \quad (2)$$

$$Z = y_3 + a = \bar{y}_3 - a = a - z_1 = \bar{z}_1 - a. \quad (3)$$

The following quantities are defined:

$$R^2 = X^2 + Y^2 + (Z-a)^2, \quad \bar{R}^2 = X^2 + Y^2 + (Z+a)^2, \quad (4)$$

$$F = -\arctan\frac{Y}{X} - \arctan\frac{Y}{Z-a} - \arctan\frac{YR}{X(Z-a)}, \quad (5)$$

$$\bar{F} = -\arctan\frac{Y}{X} + \arctan\frac{Y}{Z+a} + \arctan\frac{Y\bar{R}}{X(Z+a)}. \quad (6)$$

Each component of the displacement vector is a sum of two terms, $u_j = u_j^\infty + u_j^c$ $(j = 1,2,3)$. The first term is the displacement field of the dislocation in the infinitely extended material plus its image, and the second term releases the residual stress at the surface. The displacements are written separately for each component of the Burgers vector $\mathbf{B} = (B_1, B_2, B_3)$. The B_2 and B_3 components of the Burgers vector correspond to the edge and the screw threading dislocation arms involved in the calculations of the present paper, and the corresponding formulae for B_1 are included for a sake of completeness.

Burgers vector (B$_1$,0,0)

$$\frac{8\pi(1-\nu)}{B_1}u_1^\infty = 2(1-\nu)(F+\bar{F}) - XY\left\{\frac{1}{R\left(R-(Z-a)\right)} + \frac{1}{\bar{R}\left(\bar{R}+Z+a\right)}\right\} \tag{7}$$

$$\frac{8\pi(1-\nu)}{B_1}u_2^\infty = (1-2\nu)\left\{\log\left(R-(Z-a)\right) + \log\left(\bar{R}+Z+a\right)\right\} - Y^2\left\{\frac{1}{R\left(R-(Z-a)\right)} + \frac{1}{\bar{R}\left(\bar{R}+Z+a\right)}\right\} \tag{8}$$

$$\frac{8\pi(1-\nu)}{B_1}u_3^\infty = Y\left\{\frac{1}{R} - \frac{1}{\bar{R}}\right\} \tag{9}$$

$$\frac{4\pi(1-\nu)}{B_1}u_1^c = -\frac{(1-2\nu)XY}{\left(\bar{R}+Z+a\right)^2}\left(\nu+\frac{a}{\bar{R}}\right) + \frac{YZ}{\bar{R}\left(\bar{R}+Z+a\right)}\left\{\frac{X}{\bar{R}+Z+a}\left(2\nu+\frac{a}{\bar{R}}\right) + \frac{aX}{\bar{R}^2}\right\} \tag{10}$$

$$\begin{aligned}\frac{4\pi(1-\nu)}{B_1}u_2^c =& -\nu(1-2\nu)\log\left(\bar{R}+Z+a\right) - \frac{1-2\nu}{\bar{R}+Z+a}\left\{\nu(Z+a) - a + \frac{Y^2}{\bar{R}+Z+a}\left(\nu+\frac{a}{\bar{R}}\right)\right\}\\ &+\frac{Z}{\bar{R}+Z+a}\left\{-2\nu - \frac{a}{\bar{R}} + \frac{Y^2}{\bar{R}\left(\bar{R}+Z+a\right)}\left(2\nu+\frac{a}{\bar{R}}\right) + \frac{aY^2}{\bar{R}^3}\right\}\end{aligned} \tag{11}$$

$$\frac{4\pi(1-\nu)}{B_1}u_3^c = \frac{2(1-\nu)Y}{\bar{R}+Z+a}\left(2\nu+\frac{a}{\bar{R}}\right) + \frac{YZ}{\bar{R}}\left(\frac{2\nu}{\bar{R}+Z+a} + \frac{a}{\bar{R}^2}\right) \tag{12}$$

Burgers vector (0,B$_2$,0)

$$\begin{aligned}\frac{8\pi(1-\nu)}{B_2}u_1^\infty =& -(1-2\nu)\left\{\log(R-(Z-a)) + \log(\bar{R}+Z+a)\right\} + x^2\left\{\frac{1}{R(R-(Z-a))} + \frac{1}{\bar{R}(\bar{R}+Z+a)}\right\}\\ &-\frac{(Z-a)}{R} + \frac{(Z+a)}{\bar{R}}\end{aligned} \tag{13}$$

$$\frac{8\pi(1-\nu)}{B_2}u_2^\infty = 2(1-\nu)(F+\bar{F}) + XY\left\{\frac{1}{R\left(R-(Z-a)\right)} + \frac{1}{\bar{R}\left(\bar{R}+Z+a\right)}\right\} - Y\left\{-\frac{Z-a}{R\left(R-X\right)} + \frac{Z+a}{\bar{R}\left(\bar{R}-X\right)}\right\} \tag{14}$$

$$\frac{8\pi(1-\nu)}{B_2}u_3^\infty = -(1-2\nu)\left\{\log(R-X) - \log(\bar{R}-X)\right\} - X\left(\frac{1}{R} - \frac{1}{\bar{R}}\right) + \frac{(Z-a)^2}{R(R-X)} - \frac{(Z+a)^2}{\bar{R}(\bar{R}-X)} \tag{15}$$

$$\begin{aligned}\frac{4\pi(1-\nu)}{B_2}u_1^c =& \nu(1-2\nu)\log\left(\bar{R}+Z+a\right) + \frac{1-2\nu}{\bar{R}+Z+a}\left\{\nu(Z+a) - a + \frac{X^2}{\bar{R}+Z+a}\left(\nu+\frac{a}{\bar{R}}\right)\right\}\\ &+\frac{Z}{\bar{R}+Z+a}\left\{2\nu + \frac{a}{\bar{R}} - \frac{X^2}{\bar{R}(\bar{R}+Z+a)}\left(2\nu+\frac{a}{\bar{R}}\right) - \frac{aX^2}{\bar{R}^3}\right\}\end{aligned} \tag{16}$$

$$\frac{4\pi(1-\nu)}{B_2}u_2^c = \frac{(1-2\nu)XY}{\left(\bar{R}+Z+a\right)^2}\left(\nu+\frac{a}{\bar{R}}\right) + \frac{YZ}{\bar{R}\left(\bar{R}+Z+a\right)}\left\{-\frac{2\nu X}{\bar{R}+Z+a} - \frac{aX}{\bar{R}}\left(\frac{1}{\bar{R}} + \frac{1}{\bar{R}+Z+a}\right)\right\} \tag{17}$$

$$\begin{aligned}\frac{4\pi(1-\nu)}{B_2}u_3^c =& -\frac{2(1-\nu)X}{\bar{R}+Z+a}\left(2\nu+\frac{a}{\bar{R}}\right) + \frac{2(1-\nu)a(Z+a)}{\bar{R}\left(\bar{R}-X\right)} + \frac{Z}{\bar{R}}\left\{-\frac{2\nu X}{\bar{R}+Z+a} - \frac{aX}{\bar{R}^2}\right\}\\ &-\frac{aZ}{\bar{R}\left(\bar{R}-X\right)}\left[-\frac{(Z+a)^2}{\bar{R}^2} - \frac{(Z+a)^2}{\bar{R}\left(\bar{R}-X\right)}\right]\end{aligned} \tag{18}$$

Burgers vector (0,0,B$_3$)

$$\frac{8\pi(1-\nu)}{B_3}u_1^\infty = Y\left\{\frac{R-X}{R(R-X)}+\frac{\bar{R}-X}{\bar{R}(\bar{R}-X)}\right\} \tag{19}$$

$$\frac{8\pi(1-\nu)}{B_3}u_2^\infty = (1-2\nu)\left\{\log(R-X)+\log(\bar{R}-X\right\} - Y^2\left\{\frac{1}{R(R-X)}+\frac{1}{\bar{R}(\bar{R}-X)}\right\} \tag{20}$$

$$\frac{8\pi(1-\nu)}{B_3}u_3^\infty = 2(1-\nu)(F-\bar{F}) - Y\left\{\frac{Z-a}{R(R-X)}+\frac{Z+a}{\bar{R}(\bar{R}-X)}\right\} \tag{21}$$

$$\frac{4\pi(1-\nu)}{B_3}u_1^c = \frac{(1-2\nu)Y}{\bar{R}+Z+a}\left(1-\frac{a}{\bar{R}}\right) - \frac{YZ}{\bar{R}}\left(\frac{a}{\bar{R}^2}+\frac{1}{\bar{R}+Z+a}\right) \tag{22}$$

$$\frac{4\pi(1-\nu)}{B_3}u_2^c = (1-2\nu)\left\{-\log(\bar{R}-X) - \frac{X}{\bar{R}+Z+a}\left(1+\frac{a}{\bar{R}}\right)+\frac{Z+a}{\bar{R}-X}\frac{a}{\bar{R}}\right\} + \frac{XZ}{\bar{R}}\left(\frac{a}{\bar{R}^2}+\frac{1}{\bar{R}+Z+a}\right)$$
$$-\frac{Z}{\bar{R}-X}\left\{-\frac{a}{\bar{R}}+\frac{Z+a}{\bar{R}}\left(1+\frac{a(Z+a)}{\bar{R}^2}\right)+\frac{a(Z+a)^2}{\bar{R}^2(\bar{R}-X)}\right\} \tag{23}$$

$$\frac{4\pi(1-\nu)}{B_3}u_3^c = 2(1-\nu)\left\{\bar{F}+\frac{Y}{\bar{R}-X}\frac{a}{\bar{R}}\right\} + \frac{YZ}{\bar{R}(\bar{R}-X)}\left\{1+\frac{Z+a}{\bar{R}-X}\frac{a}{\bar{R}}+\frac{a(Z+a)}{\bar{R}^2}\right\} \tag{24}$$

III. DISPLACEMENT FIELD OF A STRAIGHT DISLOCATION PERPENDICULAR TO THE SURFACE

We present here expressions for the displacement fields of the edge and screw straight dislocations perpendicular to the surface of an elastic half-space, see Eqs. (213) and (220) in Ref. [3]. The sums of these displacement fields and those above for the angular dislocations with the Burgers vectors $(0, B_2, 0)$ and $(0, B_3, 0)$ give the L-shaped dislocation in Fig. 1(a). To do this, the direction of the Z-axis is reversed with respect to Ref. [3], and the displacement field for the edge dislocation is rotated by 90°. We define

$$r^2 = X^2 + Y^2 + Z^2. \tag{25}$$

Then,

Burgers vector (0,B$_2$,0)

$$\frac{4\pi(1-\nu)}{B_2}u_1 = (1-2\nu)\log\sqrt{X^2+Y^2} + \frac{Y^2}{X^2+Y^2} - \nu\left[(1-2\nu)\log(r+Z) + (3-2\nu)\left(\frac{Z}{r+Z}+\frac{X^2}{(r+Z)^2}\right) - \frac{2X^2}{r(r+Z)}\right] \tag{26}$$

$$\frac{4\pi(1-\nu)}{B_2}u_2 = 2(1-\nu)\arctan\frac{Y}{X} - \frac{XY}{X^2+Y^2} - \nu\left[(1-2\nu)\frac{XY}{(r+Z)^2} - \frac{2XYZ}{r(r+Z)^2}\right] \tag{27}$$

$$\frac{2\pi(1-\nu)}{B_2}u_3 = \nu X\left[\frac{1}{r} + (1-2\nu)\frac{1}{r+Z}\right] \tag{28}$$

Burgers vector (0,0,B$_3$)

$$\frac{2\pi}{B_3}u_1 = -\frac{Y}{r+Z},\quad \frac{2\pi}{B_3}u_2 = \frac{X}{r+Z},\quad \frac{2\pi}{B_3}u_3 = \arctan\frac{Y}{X} \tag{29}$$

IV. X-RAY DIFFRACTION INTENSITY IN THE STOKES-WILSON APPROXIMATION

Stokes and Wilson [4] showed that the X-ray diffraction intensity distribution in a highly distorted crystal is equal to the probability density distribution of the strain. The lim-

its of applicability of this approximation were considered in Ref. [5]. It was shown that the Stokes-Wilson approximation is applicable as long as the long range order is not seen as a coherent peak in the diffraction profiles. The relevant strain components depend on the diffraction geometry. Stokes and Wilson developed their approximation for powder diffraction, in which case the normal strain in the direction of the diffraction vector is involved. The corresponding components for the reciprocal space maps and skew diffraction geometry of singe crystals are derived below.

A. Reciprocal space maps

A general expression for the X-ray diffraction intensity from a crystal containing lattice defects is

$$I(\mathbf{q}) = \iint G(\mathbf{r}_1, \mathbf{r}_2) \exp\left[i\mathbf{q}\cdot(\mathbf{r}_2 - \mathbf{r}_1)\right] d\mathbf{r}_1 d\mathbf{r}_2, \qquad (30)$$

where the integration is performed over the volume of the crystal, $\mathbf{r}_1$ and $\mathbf{r}_2$ are two points inside the crystal and $\mathbf{q}$ is a small deviation of the diffraction vector $\mathbf{Q}$ from the reciprocal lattice point. The correlation function $G(\mathbf{r}_1, \mathbf{r}_2)$ is

$$G(\mathbf{r}_1, \mathbf{r}_2) = \langle \exp\{i\mathbf{Q}\cdot[\mathbf{U}(\mathbf{r}_2) - \mathbf{U}(\mathbf{r}_1)]\}\rangle, \qquad (31)$$

where $\mathbf{U}(\mathbf{r}_1)$ and $\mathbf{U}(\mathbf{r}_2)$ are total displacements at these points due to all defects in the crystal and $\langle\ldots\rangle$ denotes a statistical average over the distribution of the defects.

For an epitaxial film with the z-axis along the film normal, the correlation function can be written as $G(x_2 - x_1, y_2 - y_1, z_1, z_2)$, due to a translational invariance in the lateral plane (but not in the z-direction). We consider reciprocal space maps measured in a standard triple-crystal laboratory X-ray diffractometer. The incident and diffracted X-rays are collimated in the scattering plane (q_x, q_z) and integrated over the wave vector component q_y normal to it (the "vertical divergence" in a standard diffractometer setup with horizontal scattering plane). The integration of the q_y-dependent term $\exp\left[iq_y\,(y_2 - y_1)\right]$ in Eq. (30) over q_y in the infinite limits gives a delta function $\delta\,(y_2 - y_1)$, and the integral (30) is simplified to

$$I(q_x, q_z) = \int_{-\infty}^{\infty} dx \iint_0^t dz_1\, dz_2\, G\,(x, z_1, z_2) \times \exp\left[iq_x x + iq_z\,(z_2 - z_1)\right]. \qquad (32)$$

The argument $y = 0$ in the correlation function is omitted hereafter for simplicity.

For a high dislocation density, only correlations between closely spaced points are important. The difference of displacements in Eq. (31) is then approximated as $\mathbf{Q}\cdot[\mathbf{U}(\mathbf{r}_2) - \mathbf{U}(\mathbf{r}_1)] \approx \kappa_x x + \kappa_z \varsigma$, where $\varsigma = z_2 - z_1$, $\kappa_x = \partial(\mathbf{Q}\cdot\mathbf{U})/\partial x$, and $\kappa_z = \partial(\mathbf{Q}\cdot\mathbf{U})/\partial z$. Then, the statistical mean (31) can be written as

$$G(x, \varsigma, z) = \iint_{-\infty}^{\infty} P(\kappa_x, \kappa_z, z) \exp\left(i\kappa_x x + i\kappa_z\varsigma\right) d\kappa_x\, d\kappa_z. \qquad (33)$$

Here $P(\kappa_x, \kappa_z, z)$ is the joint probability distribution of the respective strain components taken at a depth z. The integral over z_1 and z_2 in Eq. (32) can be written as an integral over z and ς, and the latter integral can be extended in the infinite limits, since only small ς are relevant. Then the Fourier integral (32) gives

$$I(q_x, q_z) = \int_0^t P\left[q_x = -\frac{\partial(\mathbf{Q}\cdot\mathbf{U})}{\partial x}, q_z = -\frac{\partial(\mathbf{Q}\cdot\mathbf{U})}{\partial z}, z\right] dz. \qquad (34)$$

This equation replaces Eq. (10) in Ref. [5] where the product of probabilities is written instead of the joint probability.

The Monte Carlo implementation of Eq. (34) is straightforward. First, a pixel array is defined for $I(q_x, q_z)$ to cover the range of the wave vectors of interest. Then dislocations are generated according to their density and distribution. The sum of their displacements is used to calculate the strain at the point $(0, 0, z)$, where z is uniformly seeded from 0 to t. Since the analytical differentiation of the displacements for an angular dislocation presented above would lead to very bulky expressions, we calculate derivatives of the displacements required in Eq. (34) from a difference of the displacements at two closely spaced points. After calculating the strain components q_x and q_z in Eq. (34), 1 is added to the corresponding pixel of the array $I(q_x, q_z)$. The dislocation generation is repeated.

B. Skew diffraction geometry

Figure 2(a) reproduces a sketch of the skew diffraction geometry from Ref. [6]. The details of the geometry and the definition of the angles can be found in the cited paper. Our aim now is to average the intensity (30) over the plane perpendicular to the direction of the wave vector of the scattered wave $\mathbf{K}^{\mathrm{out}}$. Figure 2(b) shows the scattering plane (the plane containing the wave vectors of the incident $\mathbf{K}^{\mathrm{in}}$ and the diffracted $\mathbf{K}^{\mathrm{out}}$ waves). On sample rotation by an angle ω, the wave vector $\mathbf{q}$ is directed perpendicular to $\mathbf{Q}$ and has a length $q = Q\omega$. Its component along the diffracted beam direction is $q_\parallel = Q\omega\cos\theta$, where θ is the Bragg angle.

The coordinates in Fig. 2(a) are chosen so that the xz plane is perpendicular to the surface and contains the wave vector $\mathbf{K}^{\mathrm{out}}$. The wave vectors in this plane are shown in Fig. 2(c). The integration of the intensity over the plane perpendicular to $\mathbf{K}^{\mathrm{out}}$ is the integration over q_y perpendicular to this plane and the integration over $q_\perp$ in this plane. The first integration is carried out as above and gives Eq. (32) with the present choice of the axis directions. To perform the second

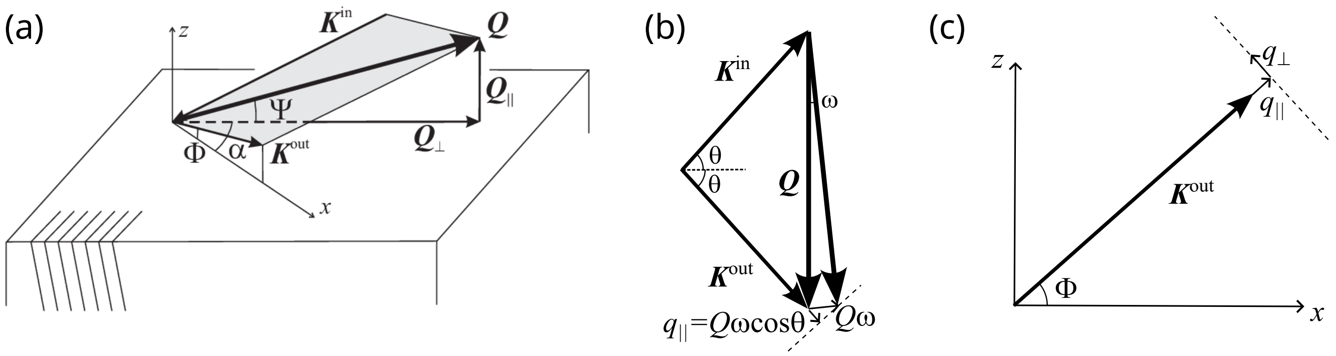

FIG. 2. Sketch of skew diffraction geometry: (a) three-dimensional picture reproduced from Ref. [6], (b) wave vectors in the scattering plane, (c) wave vectors in the plane normal to the surface and containing the scattered beam direction $\mathbf{K}^{\mathrm{out}}$.

integration, we express the wave vectors as

$$q_x = q_{\|} \cos\Phi - q_{\perp} \sin\Phi, \qquad (35)$$
$$q_z = q_{\|} \sin\Phi - q_{\perp} \cos\Phi,$$

so that

$$q_x x + q_z(z_2 - z_1) = [x\cos\Phi + (z_2 - z_1)\sin\Phi]\, q_{\|} \qquad (36)$$
$$+ [-x\sin\Phi + (z_2 - z_1)\cos\Phi]\, q_{\perp}.$$

Integrating the intensity (32) over $q_\perp$ gives rise to a delta function $\delta\left(-x\sin\Phi + (z_2 - z_1)\cos\Phi\right)$. Substituting $x = (z_2 - z_1)\cot\Phi$ in Eq. (36) gives

$$q_x x + q_z(z_2 - z_1) = q_{\|}(z_2 - z_1)/\sin\Phi, \qquad (37)$$

and the diffracted intensity is

$$I(q_{\|}) = \iint_0^t dz_1\, dz_2\, G\left((z_2 - z_1)\cot\Phi, z_1, z_2\right)$$
$$\times \exp\left[iq_{\|}(z_2 - z_1)/\sin\Phi\right]. \qquad (38)$$

This equation coincides with Eq. (6) in Ref. [7].

In the limit of threading dislocations in an infinitely thick crystal, the correlation function in Eq. (38) can be written as $G\left((z_2 - z_1)\cot\Phi\right)$, since the displacements become z-independent. Substituting $\xi = z\cot\Phi$ reduces this equation to

$$I(q_{\|}) = \int_{-\infty}^{\infty} G(\xi)\exp\left(iq_{\|}\xi/\cos\Phi\right) d\xi. \qquad (39)$$

This equation coincides with Eq. (9) in Ref. [6].

In the Stokes-Wilson approximation for Eq. (38), we write

$$\mathbf{Q}\cdot[\mathbf{U}(\mathbf{r}_2) - \mathbf{U}(\mathbf{r}_1)] \approx \frac{\partial(\mathbf{Q}\cdot\mathbf{U})}{\partial x}(z_2 - z_1)\cot\Phi$$
$$+ \frac{\partial(\mathbf{Q}\cdot\mathbf{U})}{\partial z}(z_2 - z_1) \qquad (40)$$
$$= \kappa\varsigma/\sin\Phi, \qquad (41)$$

where again $\varsigma = z_2 - z_1$ and

$$\kappa = \cos\Phi\frac{\partial(\mathbf{Q}\cdot\mathbf{U})}{\partial x} + \sin\Phi\frac{\partial(\mathbf{Q}\cdot\mathbf{U})}{\partial z}. \qquad (42)$$

Since the wave vector of the diffracted beam $\mathbf{K}^{\mathrm{out}}$ makes an angle Φ to the x axis, this equation can be written as

$$\kappa = \hat{\mathbf{K}}^{\mathrm{out}}\cdot\nabla\left(\mathbf{Q}\cdot\mathbf{U}\right), \qquad (43)$$

where $\hat{\mathbf{K}}^{\mathrm{out}}$ is the unit vector in the direction of $\mathbf{K}^{\mathrm{out}}$. The statistical average in the correlation function (31) can be written as

$$G(\varsigma, z) = \int_{-\infty}^{\infty} P(\kappa, z)\exp(\kappa\varsigma/\sin\Phi)\, d\kappa. \qquad (44)$$

The integral (38) then gives

$$I(q_{\|}) = \int_0^t P\left(q_{\|} = -\hat{\mathbf{K}}^{\mathrm{out}}\cdot\nabla\left(\mathbf{Q}\cdot\mathbf{U}\right), z\right) dz. \qquad (45)$$

For infinitely long threading dislocations, when distortions do not depend on z, this equation reduces to Eq. (2) in Ref. [8].

[1] M. Comninou and J. Dundurs, The angular dislocation in a half space, J. Elasticity 5, 203 (1975).
[2] A. L. Thomas, *POLY3D: A three-dimensional, polygonal element, displacement discontinuity boundary element computer program with applications to fractures, faults, and cavities in the Earth's crust*, Ph.D. thesis, Stanford University, USA (1993), https://purl.stanford.edu/jv679dt3587.
[3] J. Lothe, in *Elastic Strain Fields and Dislocation Mobility*, edited by V. L. Indenbom and J. Lothe (North-Holland, Amsterdam, 1992) Chap. 5.

[4] A. R. Stokes and A. J. C. Wilson, The diffraction of x rays by distorted crystal aggregates – I, Proc. Phys. Soc. London **56**, 174 (1944).

[5] V. M. Kaganer and K. K. Sabelfeld, Strain distributions and diffraction peak profiles from crystals with dislocations, Acta Cryst. A **70**, 457 (2014).

[6] V. M. Kaganer, O. Brandt, A. Trampert, and K. H. Ploog, X-ray diffraction peak profiles from threading dislocations in GaN epitaxial films, Phys. Rev. B **72**, 045423 (2005).

[7] V. S. Kopp, V. M. Kaganer, B. Jenichen, and O. Brandt, Analysis of reciprocal space maps of GaN(0001) films grown by molecular beam epitaxy, J. Appl. Cryst. **47**, 256 (2013).

[8] V. M. Kaganer, B. Jenichen, M. Ramsteiner, U. Jahn, C. Hauswald, F. Grosse, S. Fernández-Garrido, and O. Brandt, Quantitative evaluation of the broadening of x-ray diffraction, Raman, and photoluminescence lines by dislocation-induced strain in heteroepitaxial GaN films, J. Phys. D: Appl. Phys. **48**, 385105 (2015).
